# Study of antiapoptotic effect of a protein isolated from Megalopyge albicolis (Lepidoptera: Megalopygidae) hemolymph

**DOI:** 10.1186/1753-6561-8-S4-P148

**Published:** 2014-10-01

**Authors:** Marina Katia Ferreira Mazzoni, Nathalia Delazeri de Carvalho, Henrique Krambeck Rofatto, Roberto Enrique Pinto Moraes, Ronaldo Zucatelli Mendonça

**Affiliations:** 1 Instituto Butantan, São Paulo, Brazil

## 

Apoptosis has a central role in many cellular processes and development of some diseases like cancer and Alzheimer's. Molecules that interfere in the apoptotic process may be used in the biotechnology industry, the control of cell death occurring in high density cultures performed in bioreactors is an important factor in production processes. Has been shown that hemolymph of the *Lonomia obliqua *(*Lepdoptera:Saturniidade*) is capable of inhibiting cell death in different models [1-3]. The objective of this study, is to identify the potential anti-apoptotic of a protein isolated from hemolymph of larvae of *Megalopyge albicolis *(*Lepidoptera: Megalopygidae*). Methods: The hemolymph was collected and the citotoxicity was evaluated in culture (up to 5%). The anti-apoptotic protein responsible for this activity was isolated and purified by gel filtration chromatography using a gel filtration column system (Superdex 75). The fractions obtained were tested for anti-apoptotic activity in VERO and Sf-9 cells. Apoptosis was induced with 25 to 250 µM of Tert-butyl or 800ng/mL of Actinomicin D in cells treated and not treated with hemolymph. After 18 hours, the cells were stained with acridine orange and ethidium bromide and observed in confocal microscope. To study the cytoskeleton, the cells were incubated with faloidina-FITC after 4 hours of apoptosis induction. Results and Conclusions: Cytotoxicity of *Megalopyge albicolis *hemolymph was evaluated and no adverse effect was observed. This protein was capable to protect cells against death induced and was able to avoid the lost of cytoskeleton structure. The hemolymph of *M. albicolis *contain components able to inhibit death by apoptosis induced by chemical agents. Was observed that this component can act in cytoskeleton structure, increasing the cell viability acting to maintain the physiological and functional conditions of the cells. The activities exhibited by this protein are of great interest for the development of products to be employed in cell and tissue culture procedures, where the increase in cell viability is important to maintain the physiological and functional conditions of the cells.
